# MiR-145, a new regulator of the DNA Fragmentation Factor-45 (DFF45)-mediated apoptotic network

**DOI:** 10.1186/1476-4598-9-211

**Published:** 2010-08-06

**Authors:** Jianjun Zhang, Haiyan Guo, Guanxiang Qian, Shengfang Ge, Huifeng Ji, Xiaobo Hu, Wantao Chen

**Affiliations:** 1 Department of Biochemistry and Molecular Biology, Shanghai Jiao Tong University School of Medicine, Shanghai, PR China; 2 Department of Clinical Laboratory, NO.3 people's Hospital, Shanghai Jiao Tong University School of Medicine, Shanghai, PR China; 3 Department of Oral and Maxillofacial Surgery, Ninth People's Hospital, Shanghai Jiaotong University School of Medicine, Shanghai Key Laboratory of Stomatology, Shanghai, PR China

## Abstract

**Background:**

MicroRNA-145 (miR-145) is considered to play key roles in many cellular processes, such as proliferation, differentiation and apoptosis, by inhibiting target gene expression. DNA Fragmentation Factor-45 (DFF45) has been found to be the substrate of Caspase-3, and the cleavage of DFF45 by caspase-3 during apoptosis releases DFF40 that degrades chromosomal DNA into nucleosomal fragments. There are currently no in-depth studies on the relationship between miR-145 and the DFF45 gene.

**Results:**

In this study, we identified DFF45 as a novel target of miR-145. We demonstrated that miR-145 targets a putative binding site in the coding sequence (CDS) of DFF45, and its abundance is inversely associated with DFF45 expression in colon cancer cells. Using a luciferase reporter system, we found that miR-145 suppresses the expression of the luciferase reporter gene fused to the putative binding site of DFF45. The level of DFF45 protein, but not DFF45 mRNA, was decreased by miR-145, suggesting a mechanism of translational regulation. Furthermore, we demonstrate that this specific silencing of DFF45 by miR-145 accounts, at least in part, for the staurosporine-induced tumor cell apoptosis *in vitro*.

**Conclusions:**

Our study reveals a previously unrecognized function of miR-145 in DFF45 processing, which may underlie crucial aspects of cancer biology.

## Background

MicroRNAs are important post-transcriptional regulators of gene expression that control diverse physiological and pathological processes, this control allows for fine-tuning of the cellular processes, including regulation of proliferation, differentiation and apoptosis [1]. MicroRNAs are initially transcribed as long primary miRNA by RNA polymerase II or III, and cleaved sequentially by the microprocessor complex Drosha-DGCR8 to yield the precursor miRNA in the nucleus. Precursor miRNA is then exported from the nucleus and processed in the cytoplasm by Dicer. The mature miRNA is loaded together with Ago2 proteins into the RNA-induced silencing complex (RISC), where it guides RISC to silence target mRNAs through mRNA cleavage, translational repression, or deadenylation [2-4]. Most notably, changes in the abundance of a single miRNA may affect the levels of expression of hundreds of different proteins [5,6]. Although the number of verified human miRNAs is still expanding, the functions of only a few of them have been described. Recent studies have shown that the deregulation of microRNA expression contributes to the multistep processes of carcinogenesis in human cancer, either by oncogenetic or tumor suppressor function [7,8].

A putative tumor suppressing miRNA, miR-145, has been shown to be decreased in various human cancers [9-13], and it decreases the apoptosis and proliferation rate of colorectal cancer cells [14]. We have demonstrated that miR-145 targets a putative binding site in the 3'-UTR of the Friend leukemia virus integration 1 (Fli-1) gene, and its abundance is inversely related with Fli-1 expression in colon cancer tissues (data not shown). Some other targets of miR-145 include important regulators of cell apoptosis and proliferation, such as c-Myc and IRS-1 [15,16]. IRS-1, a docking protein for both the type 1 insulin-like growth factor receptor and the insulin receptor, delivers anti-apoptotic and anti-differentiation signals. MiR-145 also down-regulates the proto-oncogene c-Myc, whose aberrant expression is associated with aggressive and poorly differentiated tumors. Recently, the roles of miRNAs in cellular apoptosis have been explored widely. However, the connection between apoptotic networks and miRNA biogenesis machineries has not been investigated in depth [17-20].

In this report, we demonstrate that DFF45 expression is controlled at the translational level by miR-145, using bioinformatic and proteomic techniques. DFF45 is a caspase-3 or caspase-7 substrate that must be cleaved before apoptotic DNA fragmentation can proceed [21,22]. DFF45 exists as a heterodimer with a 40 kDa endonuclease termed DFF40, by a conserved domain of 80 amino acids at their N-terminus [23,24]. DFF45 serves as both a specific inhibitor of DFF40 and as a molecular chaperone to allow for the appropriate folding of DFF40 to become an activatable nuclease [25-27]. During apoptosis, Caspase-3 and Caspase-7-mediated cleavage of DFF45 induces the release and activation of DFF40, leading to the generation of double-stranded breaks in inter-nucleosomal chromatin regions and chromatin condensation [28]. The presence of this DNA ladder has been used extensively as a typical biochemical marker for apoptotic cell death [22,26,29]. Thus, the DFF45 may play a role in malignant transformation and metastasis, and up- or down-regulation of DFF45 expression might correlate with aggressive cancers [30,31]. By gain-of-function and loss-of function approaches, we showed that the endogenous levels of DFF45 are controlled post-transcriptionally by miR-145 in human colon cancer cells. We further investigated the function of miR-145 in apoptosis, and showed that miR-145 is necessary and sufficient to modulate the apoptotic progression through the DFF45 pathway.

## Results

### Mature miR-145 is down-regulated in colon cancer cells

**Figure 1:**
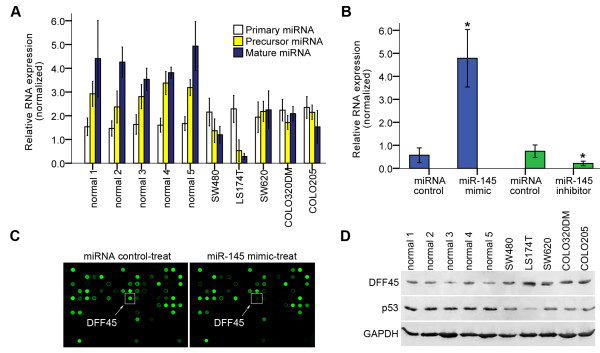
**Expression of endogenous miR-145 and DFF45 protein in colon cancer cells**. A. The expression levels of primary, precursor and mature miR-145 were examined in normal colon cells (normal), Dukes' type B cells (SW480 and LS174T), Dukes' type C cells (SW620 and COLO320DM) and Dukes' type D cells (COLO205) by real time PCR. U6 RNA was used as the quantification control. B. Detection of mature miR-145 in LS174T cells transfected with the miR-145 mimic and inhibitor. Expression of mature miR-145 in LS174T cells was quantitated 48 hours after transfection of miR-145 mimic or inhibitor by Hairpin-it™miRNAs Real-Time PCR Quantitation Assay. Assays were performed in triplicate and are shown as the mean ± SD. *: P < 0.05. C. Deregulation of the DFF45 protein was identified by antibody microarray analysis in LS174T cells transfected with the miR-145 mimic. D. Expression of endogenous DFF45 or p53 protein (wild-type or matant). Western blotting analysis was performed with total protein isolated from normal colon cells and different colon tumor cells.

We first used qRT-PCR to examine the expression of primary, precursor and mature miR-145 in normal colon cells, and in colon cancer cells at a different neoplasm staging. Compared to the normal colon cells, all cancer cells showed a significant decrease in the abundance of precursor or mature miR-145, especially in LS174T cells. However, the primary miR-145 did not change among the samples tested (Fig. 1A). We also tested the expression of wild-type p53 or mutant p53 protein in these samples, considering that it may affect the transcription or processing of miR-145 [32]. The p53 status of SW480 (Mutant p53), LS174T (Wild-type p53), SW620 (Mutant p53), COLO320DM (Mutant p53) and COLO205 (Mutant p53) has been reported previously [33]. The expression of p53 protein (wild-type p53 or mutant p53) was reduced to varying degrees in most of the colon cancer cells (Fig. 1D).

### Expression of DFF45 is inversely related to that of miR-145 in colon cancer cells

LS174T cells that express very little mature miR-145 were tranfected with a miR-145 mimic and its inhibitor. The ectopic expression of mature miR-145 was confirmed by the Hairpin-it™miRNAs Real-Time PCR Quantitation Assay. As expected, about a 6-fold increase in mature miR-145 was detected in the miR-145 mimic-transfected cells. In contrast, transfection with the miR-145 inhibitor reduced mature miR-145 by almost 50% in LS174T cells (Fig. 1B).

We then performed an antibody microarray to obtain insights into protein deregulation in LS174T cells treated with the miR-145 mimic. The five most significantly decreased proteins in the miR-145 mimic-treated group relative to the control are listed in Table 1. Among these proteins, DFF45 decreased dramatically in the cells treated with the miR-145 mimic (Fig. 1C). The other four proteins, however, were not reduced significantly after treatment with the miR-145 mimic by Western blotting (data not shown). To seek the link between miR-145 and DFF45, we measured the endogenous expression of DFF45 in normal colon cells and colon cancer cells. As shown in Figure 1D, DFF45 was overexpressed in colon cancer cells, especially in LS174T cells, in which the level of mature miR-145 was very low (Fig. 1A).

**Table 1:** The five most significantly decreased proteins in the miR-145 mimic-treated group relative to the control.

Name	Standard value (M*)	Standard value (C*)	M/C	Fold change
DFF45	0.1829	0.7215	0.2535	-3.9448
p73a	0.2419	0.8912	0.2714	-3.6841
Paxillin	0.3404	0.6529	0.5213	-1.9180
CXCR4	0.6769	1.1607	0.5832	-1.7147
Actin	0.6538	1.0844	0.6029	-1.6585

* M: miR-145 mimic-treated cells; C: miRNA control-treated cells

### MiR-145 targets a putative binding site in the coding sequence of DFF45

**Figure 2:**
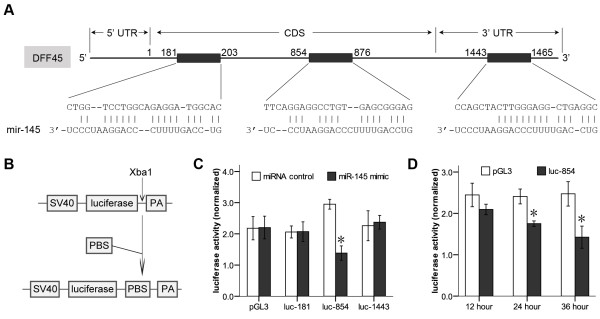
**MiR-145 targets a putative binding site in the coding sequence of DFF45**. A. Sequence alignment of miR-145 with the putative binding sites in the human DFF45 gene. The numbers are relative to the start codon site. B. Schematic diagram of the luciferase reporter construct. Putative binding sites (PBS) predicted by bioinformatics were cloned at the Xba1 site into the 3'UTR of the luciferase gene. C. Regulation of reporter gene expression by ectopic miR-145. LS174T cells were co-transfected with the miR-145 mimic and a luciferase reporter containing one putative binding site. D. Regulation of reporter gene expression by endogenous miR-145. Normal colon cells were transfected with the pGL3 vector or a luciferase reporter containing the 854-876 putative binding sites (luc-854). These experiments were performed in triplicate, and are shown as the mean ± SD, *: P < 0.05.

We used an efficient computational method (RNA22) for the prediction of the putative miR-145 binding sites in the full-length sequence of DFF45, based on minimizing the free energy of duplex structure. An alignment of human DFF45 at the predicted miR-145 binding site is shown in Figure 2A.

We chemically synthesized these putative binding sites, and tested their functions by cloning them into the Xba1 site of the pGL3 reporter vector (Fig. 2B). Using this reporter system, a functional putative binding site was identified by simply measuring luciferase activity. In LS174T cells, only the upstream binding site (854~876) responded to miR-145 over-expressed exogenously (Fig. 2C), and in normal colon cells endogenously over-expressing miR-145 (Fig. 2D).

### Specific targeting of the DFF45 putative binding site by miR-145

**Figure 3:**
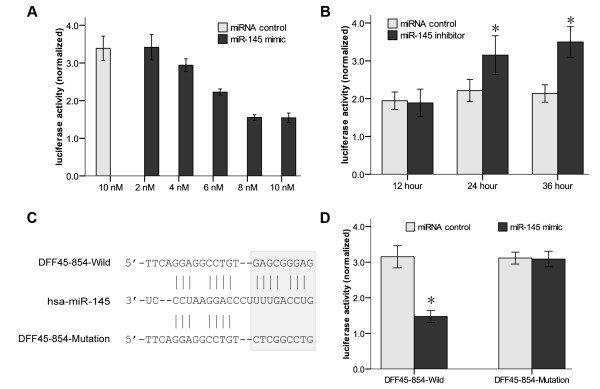
**Specific targeting of the DFF45 putative binding site by miR-145**. A. Dose-dependent suppression of luc-854 by miR-145. LS174T cells were co-transfected with luc-854 and various amounts of the miR-145 mimic. B. Effects of a miR-145 inhibitor on luciferase activity of luc-854. Normal colon cells were co-transfected with miR-145 inhibitor and luc-854. C. Schematic diagram of DFF45-854-Mutation. For the DFF45-854-Mutation, seven nucleotides (gagcGggaG) were changed with ctcgGcctG. D. Reporter mutation analysis. Downregulation of the reporter gene with the entire region (coding region plus 3'UTR) from DFF45 (DFF45-854-Wild) was apparent, whereas no effect on the DFF45-854-Mutation was detected. These experiments were performed in triplicate, and the results are shown as the mean ± SD, *: P < 0.05.

To test the specificity of miR-145 at the 854~876 site, we co-transfected LS174T cells with luc-854 and the miR-145 mimic at various abundances, and found that the inhibition of the luciferase activity by miR-145 was dose-dependent (Fig. 3A). In normal colon cells transfected with the miR-145 inhibitor, the luciferase activity was increased significantly compared to the inhibitor control at 24 hours and 36 hours (Fig. 3B).

To further demonstrate the importance of the putative binding site (854~876), a substitution mutation was generated to test its activity. In the DFF45-854-Mutation vector, seven nucleotides (gagcGgga) were replaced with ctcgGcct (Fig. 3C). We cloned the entire region (coding region plus 3'UTR) of DFF45 downstream of the reporter. As expected, down-regulation of reporter activity was detected in the construct that contains the entire region of DFF45. Correspondingly, we demonstrated that the mutation in the putative binding site (854~876) abolished the miR-145-mediated inhibition of the reporter gene (Fig. 3D). Taken together, these data suggest that the miR-145 binding site (854~876) present in the DFF45 is critical for miR-145-mediated gene regulation.

### MiR-145 regulates DFF45 at the translational level

**Figure 4:**
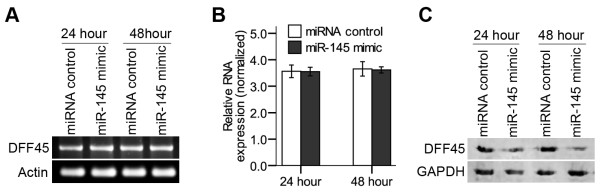
**Regulation of endogenous DFF45 expression by miR-145**. DFF45 mRNA levels in LS174T cells transfected with miR-145 mimic did not change significantly, by quantitative RT-PCR **(A) **or real-time PCR assay **(B)**, at either 24 h or 48 h after transfection. **(C) **The DFF45 protein expression levels was affected significantly by miR-145. LS174T cells were transfected with the miR-145 mimic or miRNA control. DFF45 protein levels were analyzed by Western blotting at either 24 h or 48 h after transfection. All experiments were performed in triplicate and are shown as the mean ± SD.

To identify whether DFF45 potentially regulated by miR-145, we measured the expression levels of DFF45 by quantitative polymerase chain reaction and Western blotting after treatment with the miR-145 mimic in LS174T cells. Ectopic expression of miR-145 significantly reduced the level of DFF45 protein at 24 hours and 48 hours (Fig. 4C). However, we did not detect the inhibition of DFF45 at the mRNA level, as measured by qRT-PCR (Fig. 4A) and real-time PCR (Fig. 4B). These results suggest that miR-145 targets DFF45 by functioning at the level of translational regulation.

### Detection of apoptosis by DNA fragmentation

**Figure 5:**
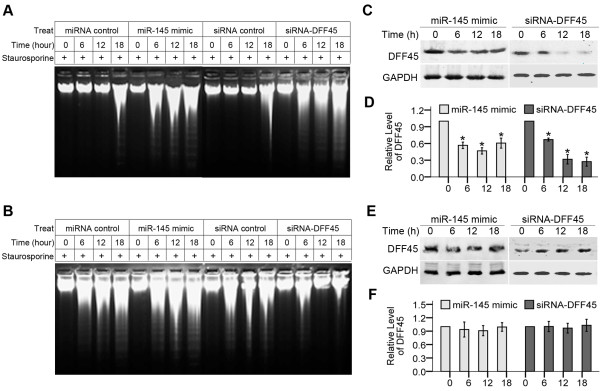
**Effects of miR-145 on staurosporine-induced DNA fragmentation**. LS174T cells **(A)**, or normal colon cells **(B) **were transfected with the miR-145 mimic or siRNA-DFF45 (50 nM), and then exposed to staurosporine. DNA ladders in samples were collected at various times after treatment with staurosporine and visualized on a 1.5% agarose gel. After transfection with the miR-145 mimic or siRNA-DFF45, down-regulation of DFF45 protein was detected by Western blotting at 6, 12 and 18 hours in LS174T cells **(C)**, but not in normal colon cells **(E)**. Values in **D **and **F **are the means of three separate experiments ± SD. *, P < 0.05.

DNA fragmentation is the typical biochemical index of cell apoptosis. These ladders of DNA fragments are the size of integer multiples of the length of a nucleosome (180-200 bp) [34]. In DNA ladder assays, cells transfected with miR-145 mimic/siRNA-DFF45 (50 nM) were exposed to staurosporine. DNA isolated from LS174T cells showed the characteristic ''ladder'' pattern of apoptosis in a time-dependent manner (Fig. 5A). As time went on, the ladder showed up more obviously in the miR-145 mimic/siRNA-DFF45-treated group. However, the time-dependent changes were not seen in DNA samples extracted from normal colon cells treated with the miR-145 mimic (Fig. 5B). To further understand the mechanisms underlying this phenomenon, we also measured by Western blotting the expression levels of DFF45 protein isolated from LS174T cells (Figs. 5C, D), or normal colon cells (Figs. 5E, F) transfected with the miR-145 mimic/siRNA-DFF45. In colon cancer cells, but not in normal colon cells, the miR-145 mimic or siRNA-DFF45 negatively regulates DFF45 expression during apoptotic progression. Non-malignant colon cells are not apparently affected by the ectopic expression of miR-145, consistent with its high level of expression in normal colon cells (Fig. 1A).

### Morphology of apoptosis detected by Hoechst staining

**Figure 6:**
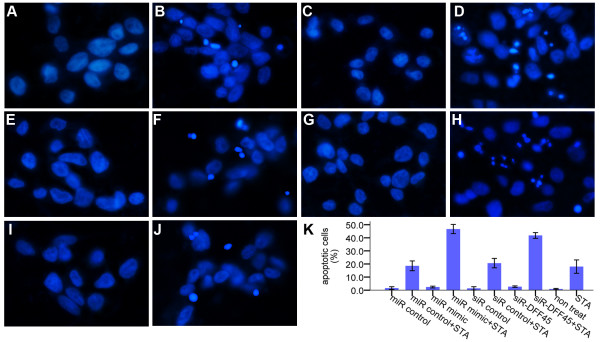
**Nuclear staining of LS174T cells with Hoechst 33258**. After transfection with the miR-145 mimic or siRNA-DFF45 (50 nM), and subsequent exposure to staurosporine (STA) for 12 h, LS174T cells were stained with Hoechst 33258, and visualized using a fluorescence microscope. **(A) **MiRNA control-treated group. **(B) **MiRNA control with STA-treated group. **(C) **MiR-145 mimic-treated group. **(D) **MiR-145 mimic with STA-treated group. **(E) **siRNA control-treated group. **(F) **siRNA control with STA-treated group. **(G) **siRNA-DFF45-treated group. **(H) **siRNA-DFF45 with STA-treated group. **(I) **Untreated group. **(J) **STA-treated group. LS174T cells treated with the miR-145 mimic or siRNA-DFF45 showed apoptotic morphology: chromatin condensation. **(K) **The percentage of cell death was calculated by counting the number of cells with condensed chromatin over the total number of cells. The data represent the mean ± SD of three independent experiments. At least 300 cells were counted for each condition.

One of the events in apoptosis is the condensation of nuclear chromatin. After being exposed to staurosporine for 12 h, the morphology of LS174T cells was investigated by Hoechst 33528 dye staining and visualization under a fluorescent microscope. Hoechst dye binds to the AT rich regions of double stranded DNA and exhibits enhanced fluorescence. Cells treated with the miR-145 mimic/siDFF45 displayed the typical apoptotic nuclear morphology (DNA condensation) (Figs. 6D, H), whereas the nuclear morphology was intact and normal in the controls. The percentage of cell death was calculated by counting the number of cells with condensed chromatin among the cells (Fig. 6K).

## Discussion

Given the great importance of DFF45 in apoptotic networks, it is reasonable to propose that a proper expression level of DFF45 will be required to achieve sensitivity to drug-induced apoptosis, and that up- or down-regulation of DFF45 expression might correlate with cancer aggression. Induction of DFF45 seems to be involved in the production of heterogenous subclones in human gastric cancer cells, and in their enhanced ability to avoid apoptosis [35]. Hara et al. found that when DFF45 is overexpressed in human renal cell carcinoma cells, it renders them highly resistant to therapy-induced apoptosis [36,37]. Additionally, thymocytes from DFF45 mutant mice exhibit neither DNA laddering nor chromatin condensation when exposed to apoptotic stimuli [38,39]. DFF45 was expressed preferably in low-stage neuroblastoma tumors, and to a lesser degree in high-stage neuroblastomas [40]. However, the molecular mechanism resulting in aberrant expression of human DFF45 in cancer cells is poorly understood.

In this report, we show that DFF45 is a direct target for miR-145. Our studies indicated that the levels of mature miR-145 were significantly lower in colon cancer cells compared with their levels in normal colon cells. Antibody microarray and Western blotting analyses on suitably prepared cell extracts showed that DFF45 levels in colon cancer cells far exceed the levels exhibited by normal colon cells (Fig. 1). There may have been a relationship between these differences in DFF45 levels and miR-145 levels. Based on these results, we selected LS174T cells for further studies. Using a luciferase reporter system, we identified a putative binding site in the CDS of human DFF45 for miR-145 (Figs. 2, 3). In LS174T cells, the miR-145 can negatively regulate DFF45 expression at the translational level (Fig. 4). The importance of miR-145 in this response was confirmed by transfection of the miR-145 mimic into LS174T cells, and the restoration of DNA fragmentation or chromatin condensation to levels similar to that of normal colon cells (Figs. 5, 6). Further, on the basis of the modest protein silencing observed in these studies, miR-145 may act to fine-tune protein expression rather than acting as an all-or-none switch. These results provided a mechanism for how the regulation of DFF45 signaling causes cancer cells to become sensitive to drug-induced apoptosis.

We also tested the expression of p53 protein that is lost or mutated in more than half of all human cancers [32]. p53 is a transcription factor that induces the expression of miR-145 by interacting with a potential p53 response element in the miR-145 promoter [15]. Additionally, in response to DNA damage, p53 interacts with the Drosha processing complex, and facilitates the processing of primary miR-145 to precursor miR-145 [41]. It is possible that the loss of p53 function may fail to stimulate miR-145 expression. Consistently, precursor miR-145 or mature miR-145 was decreased in all colon tumor cells tested, all of which had down-regulated wild-type or mutant p53 protein (Fig. 1). Based on these results, an appealing hypothesis to explain the miR-145 suppression observed in colon cancer cells is that it is linked to a deficit in miRNA processing, and there is no relation between processing of primary miR-145 to precursor miR-145 and the p53 status (wild-type or mutant).

Together, our results define the role of miR-145 in the posttranscriptional regulation of DFF45, and suggest that miR-145 provides a possible link between p53 and DFF45 in this gene regulatory network. The potential use of a natural miRNA to sensitize cells to execute full-blown apoptosis is exciting, and will hopefully lead to a new therapeutic strategy for the treatment of colon cancer.

## Conclusions

Our study revealed a previously unrecognized function of miR-145 in DFF45 processing; this function may underlie crucial aspects of cancer biology. This function may provide the possibility that the effect of chemotherapeutics for human colon cancer may be improved by utilization of miR-145 in the near future.

## Methods and materials

### Tumor cells and materials

Human colon cancer cells SW480 (ATCC Number: CCL-228™), LS174T (ATCC Number: CL-188™), SW620 (ATCC Number: CCL-227™), COLO320DM (ATCC Number: CCL-220™) and COLO205 (ATCC Number: CCL-222™) were obtained from American Type Culture Collection. Normal colon cells were collected at Renji hospital, Shanghai, China. Fresh tissue samples were immediately put into liquid nitrogen, followed by primary culture in DMEM high glucose medium containing antibiotics. MiR-145 mimic/inhibitor was purchased from Ambion (Austin, TX). SYBR Premix Ex Taq™(perfect Real Time) was obtained from Takara Bio (Madison, WI). DFF45 antibody and p53 antibody were purchased from ProteinTech Group Inc (Chicago, IL). SiRNA for DFF45 and control siRNA were purchased from GenePharma (Shanghai, China). Staurosporine was purchased from Sigma (Milwaukee, WI).

### Cell transfection

Transfection of cells was performed with Lipofectamine 2000 Reagent (Invitrogen, Carlsbad, CA) following the manufacturer's protocol. Briefly, the cells were seeded in 6-well plates at 30% confluence the day before transfection. MiR-145 mimic/inhibitor and miRNA control (50 nM each), were used for each transfection.

### Antibody Microarray analysis

Forty-eight hours after transfection with miR-145 mimic, total protein was isolated from LS174T cells and measured using BCA Protein Assay Reagent (Pierce). A human Antibody Microarray-720 slides kit was purchased from SPRING BIOSCIENCE. Briefly, the membranes were blocked with a blocking buffer, and then 0.1 mg Biotin Labeled Protein Sample was added and incubated at room temperature for 2 h. The membranes were washed, and 1 ml of Streptavidin Solution was added and incubated at room temperature for 45 min. The membranes were incubated with 1 ml of Detection Antibody-Cy3 at room temperature for 45 min. The slides were exposed to film and processed by autoradiography.

### MicroRNA and mRNA detection

QRT-PCR assays were performed for measurement of the expression levels of primary, precursor and mature miRNAs. Briefly, total RNA was extracted with a mirVana miRNA Isolation Kit (Ambion) and subjected to reverse transcription with the Reverse Transcription kit (Promega). QRT-PCR was performed with the Rotogene 3000 real time PCR system. For detection of mature miRNAs, Hairpin-it™miRNAs Real-Time PCR Quantitation Kit (GenePharma, China) was used in accordance with the manufacturer's protocol. Results were normalized to U6 snRNA. For measurement of the primary and precursor miRNA expression, real-time PCR was performed using the SYBR method and β-actin RNA was used for normalization. The primer sequences used were pre-miR-145 forward 5'-GTCCA GTTTT CCCAG GAATC-3', reverse 5'-AGAAC AGTAT TTCCA GGAAT-3'; pri-miR-145 forward 5'-TGGAT TTGCC TCCTT CCCA-3', reverse 5'-TTGAA CCCTC ATCCT GTGAG CC-3'; β*-actin *forward 5'-TCACC CACAC TGTGC CCATC TACGA-3', reverse 5'-CAGCG GAACC GCTCA TTGCC AATGG-3'; U6 snRNA forward 5'-CTCGC TTCGG CAGCA CA-3', reverse 5'-AACGC TTCAC GAATT TGCGT-3' [41].

To detect relative levels of DFF45 mRNA, real-time PCR was performed using the SYBR method at the following conditions: 95°C 30 s, 1 cycle; 95°C 5 s, 55°C 20 s, 72°C 15 s, 40 cycles. PCR primers were DFF45 forward 5'-GTTGC CTTGA ACTGG GACA-3', reverse 5'-CGCTG CTGCT ATGTG GG-3'.

### Bioinformatic prediction of miR-145 targets

Putative miR-145 binding sites in DFF45 genomic sequence were predicted by the RNA22 program based on minimizing folding energy and maximizing number of paired-up bases in heteroduplex http://cbcsrv.watson.ibm.com/rna22.html.

### Plasmid construction

Though bioinformatic analysis, the putative binding site of miR-145 was chemically synthesized and cloned into pGL3 control vector (Promega) at Xba1 site. To construct the DFF45-854-Wild vector, the entire region (coding region plus 3'UTR) of DFF45 was amplified from the cDNA of LS174T cells using DFF45 PCR primers: forward-primer 5'-GGTCC CACCT TGTGG AGGAT and reverse-primer 5'-TGAGA CGGAG TCTCG CTCTG TT, and then cloned into the pGL3 control vector at Xba1 site. To create the DFF45-854-Mutation vector, seven nucleotides (TTCAG GAGGC CTGT gagcG ggaG) were changed for the reporter construct.

### Luciferase assay

LS174T cells or normal colon cells were plated in triplicate wells of a 24-well plates and transfected with luciferase reporters fused with putative binding site for miR-145, and miR-145 mimic/inhibitor. Transfection efficiency was corrected by a renilla luciferase vector (pRL-CMV, Promega). The cells were harvested for luciferase assays 24 hour after transfection. The Dual-Luciferase Reporter Assay System (Promega) was used to measure the reporter activity according the manufacturer's protocol.

### Western blotting assay

Protein concentration was measured using Pierce BCA Protein Assay Reagent (Thermo-Fisher Scientific, Rockford, IL). Cell lysates (50 μg) were electrophoresed through 10% polyacrylamide gels and transferred to a NC membrane. The membrance was incubated with DFF45 antibody or p53 antibody. Secondary antibodies were labeled with IRDyes. Signals were visualized using an Odyssey Infrared Imaging System.

### Nuclear DNA fragmentation assay

LS174T cells (10^6^) and normal colon cells were treated with the indicated chemicals for appropriate time point. Cells were incubated in lysis buffer [10 mmol/l Tris, 1 mmol/l EDTA, 100 mmol/l NaCl, 5 g/l SDS, 1 μg/μl RNase A, pH 8.0] at 37°C for 30 min. At the end of incubation, proteinase K was added to a final concentration of 0.1 mg/ml and the incubation was continued at 50°C for 8 h. DNA was extracted with phenol/chloroform and precipitated with ethanol. DNA pellets were dissolved in TE buffer and analyzed on a 1.5% agarose gel with UV light after ethidium bromide staining.

### Condensed chromatin

Cells were seeded on sterile cover glasses placed in the 12-well plates. When they grew to approximately 70% confluence, cells were washed twice in ice-cold PBS (pH 7.4). After washing, the cells were fixed with 4% paraformaldehyde in PBS for 30 minutes at 4°C, washed twice with PBS and stained with Hoechst 33258 (Invitrogen) at a final concentration of 10 μg/ml at room temperature for 5 min. Nuclear morphology was then examined using an IX71fluorescent microscope (Olympus).

### Statistical analysis

All of the results are expressed as mean ± standard deviation. Statistical analysis was performed with Student's t-test for comparison of two groups. In both cases, differences with P < 0.05 were considered to be statistically significant.

## Competing interests

The authors declare that they have no competing interests.

## Authors' contributions

JZ carried out the molecular biology studies and drafted the manuscript. HG carried out the design of the study. GQ and SG performed the statistical analysis. HJ participated in its design. XH helped to draft the manuscript. WC performed a part of molecular biology studies. All authors read and approved the final manuscript.
